# Impact of ischemia duration on MRI-derived perfusion parameters in a mouse kidney transplant model

**DOI:** 10.1186/s41747-025-00675-x

**Published:** 2026-02-04

**Authors:** Felix L. Herr, Sandra Kloiber-Langhorst, Ming Ming Li, Olaf Dietrich, Robert Erdelkamp, Christoph Walz, Severin Jacobi, Ulrich Wirth, Jens Ricke, Clemens C. Cyran, Joachim Andrassy

**Affiliations:** 1 Department of Radiology, LMU University Hospital Munich, Munich, Germany; 2 Department of General, Visceral, and Transplant Surgery, LMU University Hospital Munich, Munich, Germany; 3 Institute of Pathology, LMU University Hospital Munich, Munich, Germany

**Keywords:** Cold ischemia, Kidney transplantation, Mice (inbred C57BL), Perfusion imaging, Reperfusion injury

## Abstract

**Objectives:**

Cold ischemia during kidney transplantation induces ischemia-reperfusion injury with endothelial dysfunction, capillary leak, and impaired perfusion. Its duration critically determines graft outcome. Dynamic contrast-enhanced magnetic resonance imaging (DCE-MRI) enables noninvasive assessment of renal microcirculation and may indicate ischemic injury. We evaluated the impact of ischemia duration on DCE-MRI-derived perfusion parameters in renal transplants in mice.

**Materials and methods:**

Procedures were approved by the local institutional animal care and use committee. A total of 15 C57BL/6 mice underwent kidney transplantation and were assigned to a short or prolonged cold ischemia group. DCE-MRI was performed to assess renal perfusion. Imaging was conducted at a mean of 268 ± 30 days (mean ± standard deviation) after transplantation. Perfusion parameters were calculated using the Patlak model, which provides the plasma volume fraction (v_p_), reflecting renal blood volume and perfusion, and the volume transfer constant (K^trans^), characterizing the rate of contrast agent extravasation from capillaries into the extravascular extracellular space.

**Results:**

Significant differences were observed in the K^trans^ parameter of transplanted kidneys between groups. The median K^trans^ (mL/100 mL/min) was significantly higher in the 16-h group (2.87, interquartile range 2.45–3.03) *versus* the 30-min group (0.91, 0.90–1.42; *p* = 0.008). Median v_p_ (mL/100 mL/min) was non-significantly lower in the 16-h group (21.89, 17.28–23.22) *versus* the 30-min group (29.02, 24.99–37.15; *p* = 0.151).

**Conclusion:**

Cold ischemia with 16-h duration was associated with significantly higher K^trans^ values in kidney transplants, reflecting significantly increased vascular permeability. DCE-MRI provides a sensitive tool for detecting ischemia-induced microvascular dysfunction.

**Relevance statement:**

Quantitative DCE-MRI detects microvascular injury after 16-h cold ischemia in kidney transplants in mice, supporting its potential as a noninvasive tool to assess graft integrity and guide interventions aimed at improving long-term transplant outcomes.

**Key Points:**

- The duration of ischemia critically affects endothelial integrity and perfusion characteristics in a mouse kidney transplant model.
- Prolonged 16-h ischemia leads to increased vascular permeability, indicating more severe endothelial and microcirculatory injury in transplanted kidneys.
- DCE-MRI enables sensitive detection of subtle ischemia-related microvascular alterations, supporting its value for noninvasive graft assessment.

**Graphical Abstract:**

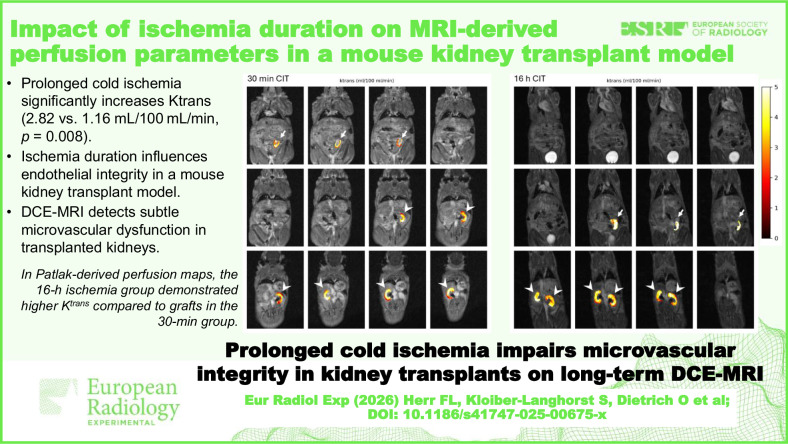

## Background

Kidney transplantation is the preferred treatment for end-stage renal disease, offering significantly longer survival and higher quality of life compared to long-term dialysis [1]. However, donor organs inevitably undergo periods of cold ischemia during procurement and transport, leading to ischemia-reperfusion injury (IRI) upon transplantation [2]. A prolonged cold ischemia time (CIT) is associated with an increased risk of acute kidney injury, delayed graft function, chronic graft dysfunction, higher rejection rates, and ultimately graft failure [3]. IRI reduces perfusion and damages the endothelium. Reoxygenation then adds hypoxia-related stress and oxidative damage [4]. Interruption of the blood supply causes endothelial dysfunction and loss of vascular integrity. During reperfusion, capillary permeability increases and edema develops. [5–7]. In parallel, there is an acute endothelial activation and vasoconstriction that can lead to an incomplete reperfusion of capillaries despite restoration of microcirculation [8]. Prolonged CIT primarily injures the renal microvasculature and tubular epithelium upon reperfusion. This underscores the importance of minimizing cold ischemia or mitigating its effects on the transplanted kidney and the use of monitoring tools to characterize graft perfusion and microvascular integrity after transplantation.

Dynamic contrast-enhanced MRI (DCE-MRI) is a noninvasive functional imaging technique for assessing renal allograft perfusion and vascular function. By capturing the first-pass kinetics of a gadolinium-based contrast agent through the kidney, DCE-MRI enables quantitative evaluation of parameters related to blood flow, capillary permeability, and vascular volume. The Patlak model yields two key metrics. The volume transfer constant (K^trans^) reflects transfer of contrast from plasma to the interstitial space and serves as a surrogate of capillary permeability. The fractional plasma volume (v_p_) represents the tissue fraction occupied by plasma and correlates with perfused blood volume [9, 10]. Together, these DCE metrics provide a characterization of renal microcirculation. Quantitative DCE-MRI approaches offer a noninvasive window into graft physiology that may help identify perfusion deficits or microvascular injury that are not apparent by conventional tests. Although ischemia-reperfusion injury has been widely studied, few investigations have quantitatively assessed the impact of cold ischemia duration on renal graft perfusion by DCE-MRI, particularly in the long-term (> 200 days after transplant).

This experimental study aimed to characterize long-term alterations induced by short (30 min) and prolonged (16 h) cold ischemia times (CIT) in transplanted murine kidneys. DCE-MRI performed approximately 200 days after transplantation was used to assess renal perfusion parameters and to determine the sensitivity of DCE-MRI in detecting microvascular sequelae of ischemia-reperfusion injury.

## Materials and methods

### Animal model and experimental protocol

All procedures were approved by the local institutional animal care and use committee (ROB-55.2-2532.Vet_02-20-186) and performed in accordance with national regulations. The study adhered to the ARRIVE guidelines [11]. Female C57BL/6J wild-type mice (Charles River), aged 8–12 weeks and weighing 20–25 g, served as both donors and recipients. Animals were housed in groups of up to four per individually ventilated cage (Type II long, Techniplast) under specific pathogen-free conditions at 22 ± 2 °C and 55 ± 10% relative humidity, with a 12-h light/dark cycle. Standard rodent chow (Altromin 1324, Altromin GmbH) and tap water were provided ad libitum. Environmental enrichment included nesting material, cardboard tunnels, and gnawing blocks. Animals were monitored daily by trained personnel for general health, body weight, and signs of distress according to predefined score sheets (provided in the Supplementary Information). Mice exhibiting pain or distress were treated according to the approved analgesia protocol or euthanized immediately if humane endpoints were reached.

**Fig. 1:**
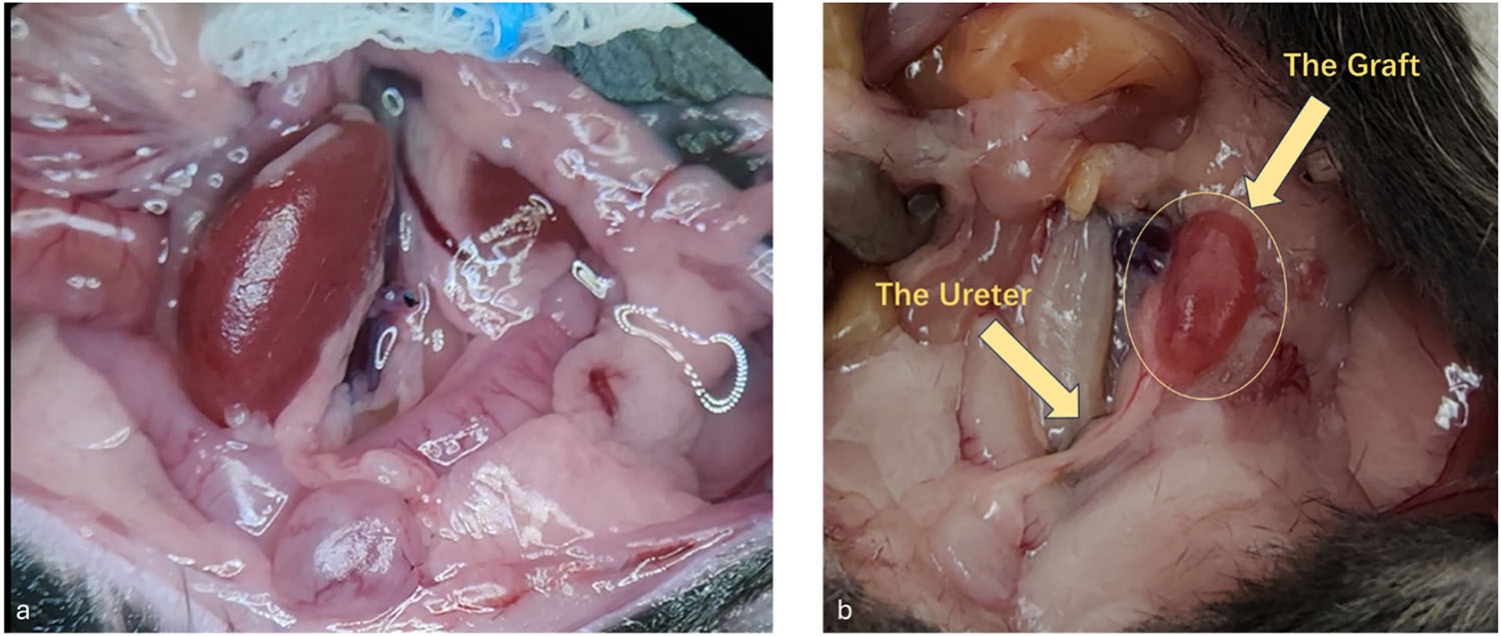
Surgical documentation of orthotopic kidney transplantation in the mouse model. **a** Intraoperative photograph showing the transplanted kidney (center) after completion of vascular anastomoses, with preserved native kidneys *in situ*. The recipient was prepared with the transplant placed orthotopically in the left lower quadrant. **b** Post-mortem macroscopic image demonstrating the anatomical positioning of the transplanted kidney (“The Graft”) and ureteral reimplantation site (“The Ureter”) in the recipient mouse. Both native kidneys were left intact throughout the experiment

Orthotopic kidney transplantation was performed under a surgical microscope using a standardized microsurgical technique. Briefly, the donor kidney, including the renal artery with a small aortic patch, renal vein, and ureter, was procured after *in situ* flushing with cold saline. In the recipient, the donor renal vessels were anastomosed end-to-side to the abdominal aorta and inferior vena cava using 11-0 nylon sutures, and ureteroneocystostomy was achieved with a pull-through technique. The surgical procedure, including orthotopic placement of the graft and preservation of native kidneys, is illustrated in Fig. 1. A detailed description of the surgical procedure is provided in the Supplementary Information.

Cold and warm ischemia times were standardized and stratified into two experimental groups. In the 30-min cold ischemia group, cold ischemia time was approximately 30 min, the time that was needed for recipient preparation. In the cold ischemia group of 16 h, donor nephrectomy was performed the evening prior to transplantation. Grafts were stored in 15 mL of Euro-Collins solution at 4 °C for approximately 16 h. Warm ischemia times were similar in both groups (~30 min).

Animals were assigned to experimental groups based on cold ischemia duration using block randomization. Blinding during MRI analysis was not feasible because graft localization had to be identified for region-of-interest segmentation. Sample size was predetermined and approved by the local institutional animal care and use committee as part of the experimental protocol, based on prior experience with similar MRI studies to achieve reliable parameter estimates while minimizing animal use.

Anesthesia was induced intraperitoneally using a combination of fentanyl (0.05 mg/kg), medetomidine (0.5 mg/kg), and midazolam (5 mg/kg) at a total injection volume of 2.5 mL/kg. Anesthesia depth was assessed via toe pinch, and mice were placed on a heated pad (Witte+Sutor GmbH) to prevent hypothermia. After surgery, anesthesia was reversed with naloxone (1.2 mg/kg), flumazenil (0.5 mg/kg), and atipamezole (2.5 mg/kg) intraperitoneally in a volume of 8.5 mL/kg. Postoperatively, mice were placed supine in warmed cages and monitored until full recovery. Buprenorphine (0.1 mg/kg) and carprofen (5–20 mg/kg) were administered every 12 h for the first 3 days. On postoperative days 4 and 5, carprofen was continued at the same dosing interval.

DCE-MRI was performed after a long-term survival period, with imaging occurring at a mean of 268 ± 29.9 days post-transplantation. The imaging time point was chosen to evaluate chronic vascular alterations rather than acute ischemia-reperfusion effects. After MRI, animals were euthanized by cervical dislocation under deep isoflurane anesthesia in accordance with institutional and governmental welfare regulations.

Only female mice were used, as group housing of males often leads to increased aggressiveness and stress. Moreover, sex-mismatched transplantations were avoided to prevent potential immunological reactions, in line with standard practice in experimental transplantation research.

The syngeneic C57BL/6J model was chosen to isolate the effects of ischemia-reperfusion injury from alloimmune responses, allowing a controlled evaluation of microvascular changes. All donor animals were immunocompetent, and their fate matched that of the recipients after transplantation.

#### MRI protocol

MRI was performed on a clinical 3-Tesla scanner (MAGNETOM Skyra, Siemens Healthineers). Under isoflurane anesthesia (2.0 vol%, 1.0 L/min O₂), mice were positioned prone in a dedicated animal cradle within a 16-channel wrist coil. Body temperature (36 ± 0.5 °C) was maintained using a heated bed and monitored via a rectal probe. Continuous physiological monitoring of heart rate and respiration was not required according to the protocol approved by the local institutional animal care and use committee. Anesthesia depth was maintained by adjusting isoflurane concentration as needed. MRI sequences included localizer scans (sagittal, coronal, axial), morphological T1- and T2-weighted gradient-echo sequences in axial and coronal planes, T1 mapping, and DCE-MRI using a dynamic time-resolved angiography with interleaved stochastic trajectories‒TWIST spoiled gradient-echo sequence with 65 repetitions (total acquisition time: 7:20 min). Imaging parameters were: repetition time 5.88 ms, echo time 2.13 ms, flip angle 19°, matrix 224 × 224 × 30, spatial resolution 0.36 × 0.36 × 1.00 mm³, orientation coronal, temporal resolution 6.6 s. Gadobutrol (Gadovist®, 1.0 mmol/mL, Bayer, Berlin, Germany) was administered intravenously at 0.2 mmol/kg body weight (1:25 dilution in saline; total injection volume ~125 µL per 25 g mouse) via a 30-G tail-vein catheter. The contrast agent was injected manually to ensure precise delivery of the small volume, as the use of an automated injector and long tubing was not suitable for this setup.

### MRI data processing

MRI images were analyzed using a clinical picture archiving and communication system workstation (Visage Imaging, San Diego, CA). Regions of interest (ROIs) of the kidneys (native and transplanted) were manually segmented in consensus by S.K.L. and C.C.C. (3 and 20 years of experience in preclinical imaging, respectively) using morphological and DCE-MRI datasets. Inter-rater reliability was not formally assessed because all measurements were performed jointly by consensus to ensure consistency. An average of 10–12 slices per kidney (covering the cortex and medulla) was included, comprising approximately 1,500–2,000 voxels per ROI. An additional ROI was placed in the abdominal aorta to extract the arterial input function. Affine motion correction was applied using in-house MATLAB-based scripts with non-rigid registration parameters. After affine motion correction, signal-time curves were calculated based on voxel-wise absolute signal enhancement (S(t) - S₀) relative to baseline, S_0_, prior to contrast agent arrival. Tracer kinetic modeling with the Patlak model was performed to extract perfusion parameters: K^trans^ (mL/100 mL/min) and v_p_ (fractional plasma volume, mL/100 mL). The Patlak model was selected because it provides robust estimates of vascular permeability and plasma volume under steady-state conditions and has been validated for analyzing extracellular contrast agents in renal DCE-MRI when backflux is negligible over the imaging time window [12]. A model-selection comparison based on the Akaike information criterion with a 2-compartment uptake model, a 2-compartment exchange model and an extended Tofts model showed that the Patlak model was most appropriate for the acquired data with relatively low temporal resolution. Median values over each ROI were used for further analysis.

### Statistical analysis

Statistical analyses were conducted using R (version 4.2.1). First, kidneys were grouped based on ischemia duration (30 min *versus* 16 h). Patlak-derived parameters (median K^trans^, v_p_) were compared between transplanted kidney *versus* native kidneys (left and right) within and across ischemia groups. Mann–Whitney *U* tests were used for unpaired comparisons (30 min *versus* 16 h), while paired Wilcoxon tests were used for intragroup analyses (transplanted kidney *versus* native kidneys). Data are presented as median and interquartile range (IQR) unless otherwise specified. For each parameter, individual data points, effect sizes (rank-biserial correlation), and 95% confidence intervals (CI) according to Hodges-Lehmann estimates [13] were reported to reflect data variability and statistical certainty. The sign of the effect size indicates directionality. A *p*-value < 0.05 was considered statistically significant. All scripts used for statistical analysis are available upon reasonable request. Due to the exploratory study design, significance levels were not adjusted for multiple comparisons.

## Results

**Fig. 2:**
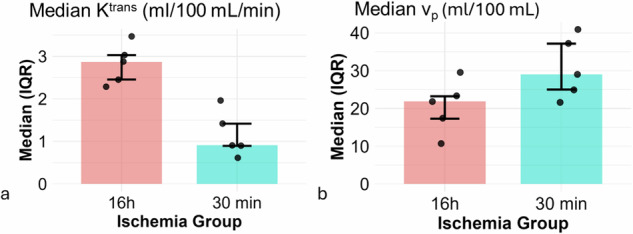
Effect of ischemia duration on Patlak-derived perfusion parameters in transplanted kidneys. Median K^trans^ in mL/100 mL/min (**a**) values were significantly higher in the 16 h ischemia group compared to the 30-min group (*p* = 0.008; effect size = 1.00; 95% confidence interval [1.04–2.26]). Median v_p_ in mL/100 mL (**b**) values tended to be lower in the 16 h group compared to the 30-min group (*p* = 0.151; effect size = -0.60; 95% confidence interval [-18.89 to 0.38]). All values are shown as median [IQR], including individual data points. Each group included *n* = 5 animals. K^trans^, volume transfer constant; v_p_, fractional plasma volume

**Fig. 3:**
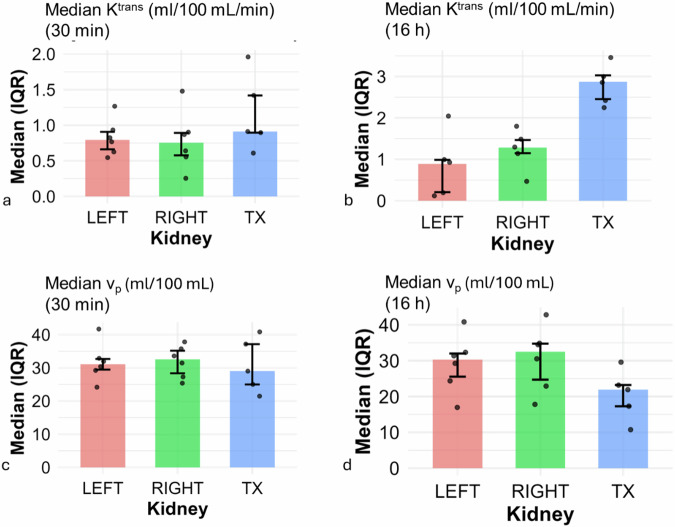
Patlak-derived perfusion parameters K^trans^ and v_p_ in the transplanted kidney (TX), left native kidney (LEFT), and right native kidney (RIGHT), stratified by ischemia duration (30-min *versus* 16 h). No statistically significant differences were observed between TX and the contralateral native kidneys for any parameter. Median K^trans^ in mL/100 mL/min tended to be higher in the TX compared to LEFT and RIGHT kidneys for both ischemia groups (**a**, **b** at 30 min and 16 h). For the TX, *n* = 5 animals were included in each group, while for the LEFT and RIGHT kidneys, *n* = 6 animals were analyzed at 30 min and *n* = 5 at 16 h. In contrast, median v_p_ in mL/100 mL tended to be lower in the TX only in the 16-h group compared to the native kidneys (**c**, **d** median v_p_ at 30 min and 16 h). For the transplanted kidneys TX, *n* = 5 animals were included, while for the native LEFT and RIGHT kidneys, *n* = 6 animals were analyzed in both groups. All values are shown as median [IQR], including individual data points. K^trans^, Volume transfer constant; v_p_, Fractional plasma volume; TX, Transplanted kidney

A total of 15 C57BL/6 mice underwent successful kidney transplantation and were assigned to either a short (30 min; *n* = 6) or prolonged (16 h; *n* = 9) cold ischemia group. Dynamic contrast-enhanced MRI measurements were successfully acquired in most animals. Due to motion artifacts and incomplete DCE-MRI acquisitions, Patlak-derived perfusion parameters could not be obtained in all animals. The number of successfully analyzed datasets corresponds to the individual data points shown in Figs. 2 and 3.

### Intergroup comparison: 30-min *versus* 16-h cold ischemia duration

A significant effect of ischemia duration was observed in the transplanted kidneys. Median K^trans^ values were significantly higher in the 16-h group compared to the 30-min group (median [IQR]: 2.87 [2.45–3.03] *versus* 0.91 [0.90–1.42] mL/100 mL/min, *p* = 0.008; effect size = 1.00; 95% CI [1.04–2.26]; Fig. 2a), indicating increased capillary permeability with 16-h ischemia. Although the v_p_ values in the transplanted kidneys tended to be lower in the 16-h group compared to the 30-min group (median [IQR]: 21.89 [17.28–23.22] *versus* 29.02 [24.99–37.15] mL/100 mL, *p* = 0.151; effect size = -0.60; 95% CI [-18.89 to 0.38]; Fig. 2b), these differences did not reach statistical significance.

### Intragroup comparison: transplanted kidney *versus* native kidneys

Within both ischemia groups, no statistically significant differences were observed between the transplanted kidney and the contralateral native kidneys (left and right) for any of the Patlak-derived parameters. In the 30 min group, median K^trans^ tended to be 0.91 [0.90 to 1.42] mL/100 mL/min in the transplanted kidney, 0.80 [0.66–0.91] mL/100 mL/min in the left (*p* = 0.313; effect size = 0.60; 95% CI [-0.32 to 0.87]) and 0.75 [0.58–0.89] mL/100 mL/min in the right kidney (*p* = 0.438; effect size = 0.20; 95% CI [-0.87 to 1.16]; Fig. 3a). In the 16-h group, median K^trans^ tended to be 2.87 [2.45–3.03] mL/100 mL/min in the transplant, 0.89 [0.21–0.99] mL/100 mL/min in the left (*p* = 0.125; effect size = 1.00; 95% CI [1.39–2.82]), and 1.29 [1.15–1.47] mL/100 mL/min in the right kidney (*p* = 0.250; effect size = 1.00; 95% CI [1.13–1.65]; Fig. 3b).

In the 30-min group, median v_p_ tended to be 29.02 [24.99–37.15] mL/100 mL in the transplant, compared to 31.12 [29.46–32.67] mL/100 mL in the left (*p* = 0.438; effect size = -0.60; 95% CI [-7.92 to 5.24]) and 32.54 [28.36–35.16] mL/100 mL in the right kidney (*p* = 0.813; effect size = 0.20; 95% CI [-8.58 to 2.99]; Fig. 3c). In the 16-h group, median v_p_ tended to be 21.89 [17.28–23.22] mL/100 mL in the transplant, 30.29 [25.55–31.99] mL/100 mL in the left (*p* = 0.625; effect size = -0.50; 95% CI [-30.07 to 6.35]), and 32.48 [24.70–34.75] mL/100 mL in the right kidney (*p* = 0.625; effect size = -0.50; 95% CI [-32.11 to 5.28]; Fig. 3d).

**Fig. 4:**
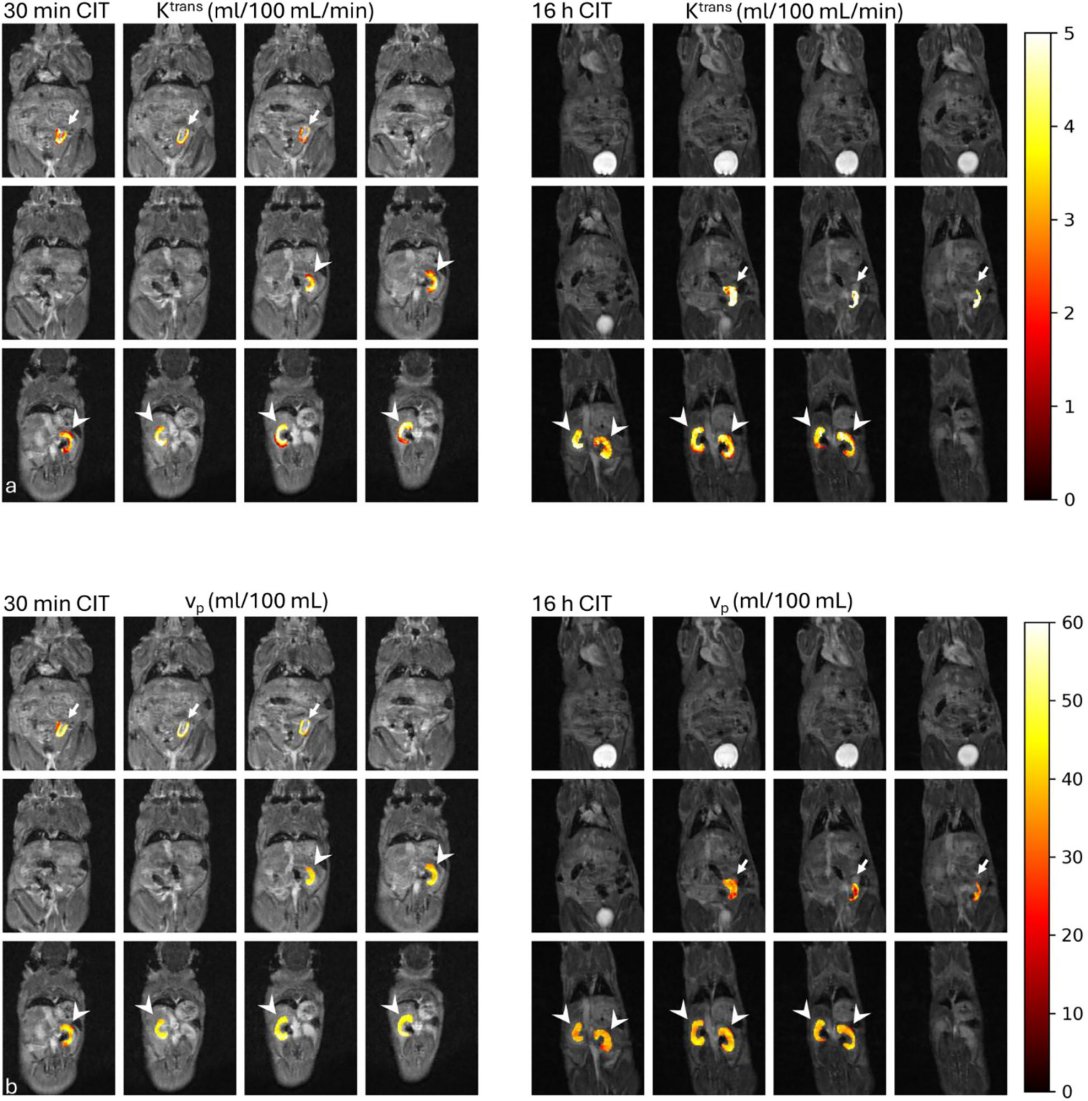
Representative coronal dynamic contrast-enhanced MRI images overlaid with Patlak-derived perfusion maps in murine kidney transplant recipients after short (30 min, left panel) or prolonged (16 h, right panel) ischemia time. **a** K^trans^ maps in mL/100 mL/min demonstrate higher values in the transplanted kidneys (white arrows) compared to native kidneys (white arrowheads) in both groups. The 16 h ischemia group demonstrated higher K^trans^ compared to grafts in the 30-min group. **b** Corresponding v_p_ maps in mL/100 mL show lower fractional plasma volumes in the 16-h ischemia group compared to 30-min ischemia in the transplanted kidneys (white arrows), indicating reduced perfused vascular volume. V_p_ maps demonstrate lower values in the transplanted kidneys compared to native kidneys (white arrowheads) for the 16-h group, in contrast, v_p_ was similar to those of the two native kidneys in the 30-min group. The color bar indicates K^trans^ and v_p_ scale ranges. CIT, Cold ischemia time; K^trans^, Volume transfer constant; v_p_, Fractional plasma volume

Figure 4 illustrates representative DCE-MRI perfusion maps (K^trans^ and v_p_) from mice in the 30-min and 16-h ischemia groups.

## Discussion

In this study, we employed DCE-MRI to evaluate renal graft perfusion after short (~30 min) and prolonged (~16 h) cold ischemia times, more than 200 days after transplantation. Compared with the 30-min group, the 16-h group showed higher K^trans^ (*p* = 0.008) and lower v_p_ (*p* = 0.151). This pattern is consistent with increased capillary permeability and a reduced perfused plasma volume after 16-h prolonged ischemia. In both ischemia groups, K^trans^ tended to be elevated in the transplanted kidneys compared to the native contralateral kidneys, suggesting increased capillary permeability following transplantation. This effect was more pronounced in the 16-h group. In the 30-min group, v_p_ was similar to that of the two native kidneys. In the 16-h group, the transplanted kidney tended to exhibit a lower v_p_, although no statistically significant differences were observed between the transplanted kidney and the contralateral native kidneys. Together, these findings support our hypothesis that extended cold ischemia damages endothelial integrity.

### Impact of ischemia duration on graft perfusion

The comparison between our 30-min and 16-h ischemia groups suggests that 16-h cold ischemia exacerbates microvascular injury and alters post-transplant perfusion. In particular, the 16-h ischemia kidneys had roughly two-fold higher K^trans^ than the 30-min ischemia kidneys, alongside a ~30% lower v_p_. The elevated K^trans^ observed in the 16-h ischemia group most likely reflects increased capillary permeability or leakiness, indicative of endothelial injury. In parallel, the reduction in v_p_ suggests a loss of functional plasma volume within the graft tissue, consistent with either decreased capillary density or ischemia-induced vasoconstriction limiting intrarenal blood volume. Although the differences in v_p_ did not reach statistical significance, likely due to the limited sample size. Our findings align with prior experimental studies on transplant ischemia-reperfusion injury. Ren et al found that longer periods of cold ischemia (from 2 to 4 h) led to progressively inhibited MRI perfusion metrics in a rat model [14]. Similarly, in a mouse transplant study, Zhang et al qualitatively observed lower DCE-MRI enhancement in grafts after 4 h ischemia compared to 0.5 h, corresponding with tissue injury in the longer-CIT grafts [15]. Our findings also align with recent clinical data. Another study reported that prolonged cold ischemia is associated with impaired graft microcirculation and altered reperfusion patterns despite the use of preservation techniques such as hypothermic machine perfusion [16]. This further supports the notion that extended ischemia induces persistent endothelial dysfunction that remains detectable well beyond the immediate reperfusion period. By comparison, our 16-h CIT condition represents an extremely prolonged ischemia, far beyond the 4–6 h range that caused acute damage in prior rodent models [15]. Therefore, the 16-h kidneys in our study, despite evidence of microvascular dysfunction, were still perfused and viable. This underscores that even when prolonged cold ischemia does not immediately destroy graft viability, it can induce microvascular derangements that may portend functional consequences. In summary, different cold ischemia durations produce different perfusion profiles post-transplant, with prolonged cold ischemia uniquely predisposing to a hyperpermeable, low-capillary-volume state in the graft. These imaging-based insights reinforce the clinical emphasis on minimizing CIT to improve transplant outcomes [3].

### Comparison of transplanted *versus* native kidney perfusion

A result of our study is that the transplanted kidneys did not significantly differ from the contralateral native kidneys in any perfusion parameter, in both the 30-min and 16-h ischemia cohorts. In the 30-min ischemia group, the transplanted kidney’s perfusion was virtually indistinguishable from the two native kidneys, indicating that a mild ischemic insult had no lasting impact on cortical microcirculation by the time of imaging. This finding suggests that, given an adequately short cold storage, the transplanted organ can reperfuse and function akin to a normal *in situ* kidney almost immediately [17]. Clinically, this corresponds to scenarios of immediate graft function, such as living-donor transplants or well-preserved deceased-donor kidneys with minimal ischemia, as these grafts often show prompt diuresis and perfusion comparable to native kidneys in the early postoperative period. Our imaging results provide a quantitative confirmation of these observations: after only 30 min of cold ischemia, the renal blood volume and contrast uptake rate in the graft were almost on par with native kidney benchmarks. This aligns with other reports showing that kidneys exposed to very short ischemia can regain nearly normal function within hours post-transplant [17], underscoring that a mild ischemic period is well tolerated if reperfusion is prompt.

In the 16-h ischemia group, we did observe trends toward perfusion differences (higher K^trans^, lower v_p_) between the transplant and native kidneys, but due to variability and limited sample size number of animals these did not reach statistical significance. The direction of the changes was consistent with expectations of IRI in the graft, and such changes were not seen in the 30-min group. One interpretation is that the transplanted kidneys were adversely affected by the prolonged cold ischemia, resulting in subtle microvascular dysfunction despite restored bulk flow [18].

This aligns with recent clinical data showing that prolonged ischemia remains difficult to fully mitigate despite perfusion advancements. For instance, a 2025 randomized trial by Dajti et al found that hypothermic oxygenated machine perfusion‒HOPE did not significantly reduce the incidence of delayed graft function compared to static cold storage, except in selected high-risk donor subsets [19]. Despite 16 h of cold storage, the transplanted kidneys still achieved substantial reperfusion. This indicates some resilience and compensatory reperfusion even after prolonged preservation. One possible reason is that in our model, the transplant was not required to sustain on its own, since the two native kidneys were left intact. Thus, the transplanted kidney in the 16-h group was reperfused into an environment of normal systemic renal function, which may have moderated the hemodynamic stress on the graft. In a clinical setting where the transplanted kidney is typically the sole functioning kidney, prolonged ischemia might have more dramatic consequences, since the entire renal blood flow must pass through the graft, and its function is immediately critical for the host.

In our scenario, the native kidneys maintain systemic homeostasis, and the transplant would still receive an adequate blood supply from the host circulation. This mirrors clinical observations that some kidneys with quite long ischemia can function if supported during the initial period, for example, with temporary dialysis support or the patient’s residual renal function. Indeed, even kidneys preserved for 30–36 h have been reported to recover and show acceptable function at 1-year post-transplant, provided they are carefully managed and supported in the interim [20]. Our findings are in line with these reports by demonstrating that prolonged cold storage, while inducing some perfusion aberrations, does not preclude a substantial reperfusion of the graft when systemic support is available. The subtle perfusion disparities we observed in the 16-h ischemia group could be clinically meaningful. Even without an overt reduction in total renal blood flow, the quality of microvascular perfusion in the transplanted kidney appears altered, with signs of leaky capillaries or uneven capillary flow, consistent with IRI-related microvascular dysfunction [21]. Such changes might predispose the graft to complications, for instance, slower clearance of waste products, focal ischemic micro-regions, or heightened susceptibility to immune-mediated injury [21, 22]. Therefore, detecting these perfusion alterations is important even when global perfusion seems adequate. The fact that our 16-h grafts still had roughly comparable bulk perfusion to native kidneys suggests that standard clinical measures, like gross renal blood flow or a single-time-point serum creatinine, might not immediately flag a problem. In contrast, functional MRI parameters can unveil the underlying microvascular stress. This highlights the potential utility of MRI in assessing graft perfusion.

### Immunological considerations in syngeneic long-term grafts

Although our model used syngeneic C57BL/6 mice, the possibility of chronic immunological injury cannot be entirely excluded. While no overt rejection was observed, the significant increase in K_trans_ at over 200 days after transplant in the 16 h ischemia group may reflect not only IRI but also subtle immune-mediated microvascular remodeling. This interpretation is supported by emerging evidence that minor histocompatibility antigen mismatches can provoke chronic injury even in genetically identical strain combinations. For example, female recipients of male syngeneic grafts mount immune responses against the H-Y antigen, leading to rejection and loss of graft function over time despite major histocompatibility complex identity [23]. Immunological monitoring and histological validation were not part of the study design and therefore were not performed. Therefore, any reference to potential immune mechanisms should be regarded as speculative rather than definitive. If any immune-mediated effects occurred, they would have affected both experimental groups equally, given the syngeneic transplantation model.

In our study, mice were followed for more than 200 days post-transplant, a timespan rarely reported in prior imaging studies. In this context, even low-level immune recognition of minor histocompatibility antigen may progressively contribute to microvascular inflammation and capillary dysfunction. Long-term syngeneic grafts have previously shown signs of glomerulosclerosis and vasculopathy in histology, attributed to non-major histocompatibility complex antigenic disparities and immune-driven endothelial injury, especially when the observation window exceeds 100 days [24]. Our elevated K^trans^ values suggest an ongoing disturbance in vascular permeability, potentially reflecting such subclinical processes. Furthermore, T cell-mediated immune surveillance and innate immune responses may sustain low-grade inflammation in long-surviving grafts. Studies have shown that such immune pressure can impair capillary integrity even without full rejection, mimicking the vascular leakage we identified via DCE-MRI [25, 26]. This is particularly relevant since perfusion deficits caused by immunological injury may not manifest as gross reductions in renal blood flow but can still alter Patlak-derived K^trans^ values due to capillary leakiness. In this light, our findings may represent not only the sequelae of ischemic injury but also the cumulative burden of immunological microvascular stress. The fact that the native kidneys remained unaffected strengthens the case that the transplant-specific microenvironment and antigenicity play a unique role in these chronic perfusion changes. Although low-grade immune effects cannot be fully excluded, our data provide no direct evidence of immunological activity. Accordingly, the observed elevation in K^trans^ values should be interpreted primarily as a marker of microvascular dysfunction consistent with ischemia-reperfusion injury, with immune contributions remaining a theoretical possibility. Future studies combining DCE-MRI with complementary histological or biochemical analyses could further substantiate these observations.

### Limitations

The study includes a relatively small sample size, especially within subgroups after exclusion of technically incomplete datasets, which limits statistical power. Some differences, such as those in v_p_, therefore remained at a trend level. Additionally, potential interindividual anatomical variations in the small mouse kidneys could influence quantification; however, we largely counteracted this through standardized ROI placements and modeling.

In this study, we used a non-life-sustaining renal transplant model in the mouse, in which both native kidneys remained *in situ* and functional after transplantation. Consequently, we cannot make a statement on the functional performance of the transplanted grafts. While this setup differs from the clinical situation of solitary kidney transplantation, it represents a fully vascularized and technically demanding experimental model that allows detailed assessment of graft perfusion and viability under controlled conditions. The presence of native kidneys may have reduced the hemodynamic and metabolic stress on the graft, potentially attenuating ischemic injury. Nevertheless, the observation that 16-h cold ischemia compromises graft perfusion and viability is consistent with clinical experience, where extended ischemia time is known to adversely affect graft quality. Therefore, our findings remain translationally informative, even though direct extrapolation to the clinical setting should be made with caution.

Finally, it should be noted that immunological reactions, even though not intended by choosing the syngeneic transplant model, could not be excluded. But this should have been similar in the two experimental groups. The study relied exclusively on MRI-derived parameters without histological, immunohistochemical, or biochemical validation. This absence of complementary outcome measures limits the ability to directly correlate imaging findings with underlying tissue pathology. Furthermore, no blood chemistry analyses (*e.g*., serum creatinine, blood urea nitrogen, or cytokine levels) were performed, which restricts functional correlation with renal physiology. In addition, no immunological assays were conducted; therefore, all references to possible immune-mediated microvascular changes should be interpreted as speculative. Future work combining imaging with histological, immunological, and biochemical endpoints is warranted to confirm these interpretations. Although no histological assessment was performed to confirm immune cell infiltration, we cannot exclude that the observed microvascular alterations, particularly in the 16-h group, may partially reflect chronic low-grade immunological responses driven by minor histocompatibility mismatches despite syngeneic transplantation. Possible interactions between prolonged ischemia and enhanced alloimmunity have been discussed in other studies [27, 28] and offer starting points for further research.

## Conclusions

In conclusion, this study provides new insights into how different cold ischemia durations affect transplanted kidney perfusion over time, using quantitative DCE-MRI. Cold ischemia of 16 h resulted in a microvascular injury signature on MRI, characterized by increased contrast transfer and decreased plasma volume, consistent with augmented capillary permeability and reduced perfused capillary density from ischemia-reperfusion injury. In contrast, a cold ischemia of 30 min led to perfusion parameters that were almost indistinguishable from native kidneys. These findings confirm our hypothesis that extended ischemia has a deleterious effect on graft microcirculation, even if bulk blood flow is re-established. DCE-MRI proved capable of detecting these subtle perfusion abnormalities after transplant. As such, quantitative MRI perfusion assessment may be a valuable addition to clinical transplant evaluation, enabling identification of grafts suffering from severe IRI.

## Supplementary information

ELECTRONIC SUPPLEMENTARY MATERIAL

## Data Availability

The datasets used and/or analyzed during the current study are available from the corresponding author on reasonable request.

## Abbreviations

CI: Confidence interval
CIT: Cold ischemia time
DCE: Dynamic contrast-enhanced
IQR: Interquartile range
IRI: Ischemia–reperfusion injury
K^trans^: Volume transfer constant
MRI: Magnetic resonance imaging
ROI: Region of interest
v_p_: Fractional plasma volume
