# Supplementary material for: Impact of ischemia duration on MRI-derived perfusion parameters in a mouse kidney transplant model
Source: Eur Radiol Exp. 2026 Feb 4;10:11. doi: 10.1186/s41747-025-00675-x (PMC12873054; doi:10.1186/s41747-025-00675-x)
Supplement: Supplementary file 1 — ELECTRONIC SUPPLEMENTARY MATERIAL [file 41747_2025_675_MOESM1_ESM.pdf]

Impact of ischemia duration on MRI-derived perfusion parameters in a mouse kidney transplant model

ELECTRONIC SUPPLEMENTARY MATERIAL

Kidney transplantation procedure

Donor nephrectomy was performed under a stereomicroscope using an established microsurgical technique. After midline laparotomy, the left kidney, including the renal artery with a small aortic patch, the renal vein, and the ureter, was mobilized by cauterizing and dividing the small lumbar and gonadal vessels. The kidney was then flushed in situ with cold saline and procured en bloc. In the recipient, both native kidneys were preserved. Following exposure of the abdominal aorta and inferior vena cava, the donor kidney was implanted orthotopically, with end-to-side anastomoses of the donor renal artery to the recipient's abdominal aorta and the donor renal vein to the inferior vena cava using continuous 11-0 nylon sutures. For urinary tract reconstruction, the ureter was introduced into the bladder using a pull-through technique. The periureteral tissue was fixed to the bladder wall with interrupted sutures, and the distal ureter was shortened to allow for proper positioning within the bladder lumen. The surgical procedure, including orthotopic placement of the graft and preservation of native kidneys, is illustrated in Fig. 1.

Burden Score Sheet (English version)

**Burden Level**	none	mild	moderate	severe
**Points**	0	5	10	20

Behavior

none (0 points): inconspicuous, normal

mild (5 points): reduced but normal upon stimulation

moderate (10 points): reduced even under stimulation, hyperkinesia or reduced motor activity

severe (20 points): apathetic, self-injury, vocalization, self-isolation

General Condition

none (0 points): coat smooth and shiny; body openings clean; eyes clear

mild (5 points): reduced or increased grooming

moderate (10 points): dull coat, piloerection, unkempt body openings, cloudy eyes

severe (20 points): dirty coat, crusted body openings, rapid shallow breathing or gasping, paralysis, tense painful abdomen

Weight Loss

0 points: 0–4%

5 points: 5–9%

10 points: 10–14%

20 points: >15%

Pain Symptoms

none (0 points): none

mild (5 points): crouching posture but normal reaction to stimulation

moderate (10 points): arched back, continuously lowered head, but normal reaction to stimulation

severe (20 points): vocalization, aggression upon stimulation, self-mutilation, crouched posture, arched back even under stimulation

Surgical Wound

none (0 points): unremarkable

mild (5 points): secretion from wound

moderate (10 points): beginning wound dehiscence

severe (20 points): wound dehiscence

Hydration

none (0 points): normal

mild (5 points): reduced

moderate (10 points): skin fold remains for 1–2 seconds

severe (20 points): skin fold remains for more than 2 seconds

Termination Criteria

If 20 points are reached in any individual category, or in the sum of multiple categories, the experiment must be terminated.

The animal is euthanized under anesthesia by exsanguination or cervical dislocation.

Instructions for Action

Mild burden:

Inform responsible personnel. Monitoring frequency every 24 hours. Interventions may be necessary (e.g., analgesia, subcutaneous fluid supplementation, soft food).

Moderate burden:

Animal must be presented to the responsible person and the veterinarian. Interventions may be necessary (e.g., analgesia, subcutaneous fluids, soft food). Monitoring frequency every 12 hours.

Severe burden:

If symptoms in the "severe" category occur, OR if 3 or more symptoms from the "moderate" category are present, this is considered a severe burden. Immediate euthanasia is required.

Postoperative Burden Classification

On the day of kidney transplantation: moderate burden. On postoperative days 2–3: mild burden. If mild burden persists or reappears after postoperative day 3, the study director or veterinarian must be contacted. New mild symptoms should resolve within 2 days; moderate symptoms within 3 days. Based on findings: prolong or repeat analgesia, or terminate the experiment.

Hydration Rule

A skin fold that remains for more than 2 seconds requires immediate fluid substitution. If hydration has not improved by the next scoring time, euthanasia must be performed.
